# The Treatment of the Summer Diarrhœa of Children

**Published:** 1893-10-28

**Authors:** Theodore Fisher


### THE TREATMENT OF THE SUMMER DIARRHŒA OF CHILDREN.

(By Theodore Fisher, M.D.Lond.)

Perhaps one of the diseases that most commonly comes under the notice of the practitioner, and yet at the same time defies all his attempts to combat it, is diarrhœa in children. In a large number of cases, in spite of repeated changes of medicinal treatment, there is a steady downward progression, ending in collapse and death. It has been frequently remarked that the longer the list of drugs named as useful in a disease the less efficacious is the medicinal treatment, an observation that is fully borne out here. Amongst the numerous remedies advocated from time to time for summer diarrhœa of children, not one can be relied upon as of real value.

Scientific treatment is based upon the knowledge of the pathology of a disease. When the pathology is clear the physician endeavours to strike at the root of the trouble, but when he can see no farther than symptoms and physical signs, he has to be satisfied with treating what he sees. In this disease, however, the treatment of symptoms is unsatisfactory, and drugs known to be useful in other forms of diarrhœa are found to be useless. While it cannot be said that treatment other than that merely directed to arrest the diarrhœa is satisfactory, yet since treatment of symptoms fails, it is necessary to endeavour to go deeper. Apparently the disease is of a specific nature, and the source of infection is probably conveyed to the body by food or drink. Chemical changes occur in the contents of the intestines, and the products of decomposition absorbed produce slow poisoning. The child becomes drowsy and finally unconscious, lying in bed with eyes half open, the cornea covered with a thin film. At the same time there is coldness of the extremities, while the internal temperature rises, sometimes reaching a great height. At this stage occasionally a general œdema of a rather curious kind sets in, the skin losing its elasticity will retain for some time any folds that may be pinched up. Within the last few years intestinal antiseptics have been commonly given, with the view of arresting the production of poisons in the intestines.

Salicylate of soda, 1 to 3 grs., every two hours; salol, ½ gr. to 1 gr. or more; liq. hydrarg. perchlor. ♏v. to ♏x.; liq. arsenicalis, ♏ ¼ to ♏j., and resorcin or naphthalin. Any of these drugs may be usefully combined with some preparation of bismuth. Although in some cases benefit may be derived from one or other of these drugs, unfortunately no great confidence can be placed in them. And this is not surprising when one thinks how small a quantity of the antiseptic can ever reach the intestine, and when it arrives there how very much it must be diluted. Yet the diminution of abdominal distension in cases of typhoid treated with intestinal antiseptics seems to show that they may be of some use. In addition to giving a drug of this character, it is deemed advisable to replace milk by some other article of diet. This is done for two reasons; firstly, because the infection is probably conveyed to the body in many cases by milk; and secondly, because, with impaired digestion, the undigested milk forms a nidus for decomposition and the production of harmful poisons. Food, therefore, is given in a form in which it is readily absorbed, hence some peptonised form of beef tea is useful. Raw meat juice, veal broth, Valentine's meat juice, Brand's essence, or barley water with white of egg beaten up in it may be given. If milk is given at all, it is better peptonised. This is readily done by the addition of some peptonising powder or Benger's liquor pepticus, and allowing the mixture to stand in a warm (but not too hot) place near the fire for about two hours. Not more should be prepared than will last half a day, since the peptonised food readily decomposes.

Since the treatment both by diet and drugs is directed towards the arrest of decomposition, it is obvious that it is advisable to commence by clearing the intestines of their contents. Some aperient should therefore be given, ¼ to ½ gr. of calomel every two hours for twelve hours, ½ dr. to 1 dr. of castor oil twice or three times repeated, or sulphate of magnesia, 5 to 10 gr., every hour for three or four doses. The condition of the pulse towards the later stages of the disease suggests that stimulants may be of value, and brandy ♏x. or more may be given according to the age of the child. Since a high atmospheric temperature appears to have a depressing effect the child must be kept in a cool and airy room. In many cases, in spite of all treatment, the case progresses from bad to worse until a collapse sets in, with cold extremities and high internal temperature. When this stage is reached, or perhaps before, a mode of treatment recommended by American writers and called enterocylsis may be of service. It consists in the irrigation of the large intestine with ice-cold water. The following is a description of the method taken from a paper by Dr. Müller, of the Jefferson Medical College, in the *Therapeutic Gazette* for July 15th, 1893. "In practising the irrigation the child is placed on a bed protected by a rubber cloth, and the catheter lubricated with vaseline is gently introduced into the anus. . . As the rectum is not a straight tube the catheter should be flexible; if a hard tube be used the sensitive mucous membrane may readily be injured. For its first inch the catheter should be introduced in the direction of the umbilicus, and then towards the hollow of the sacrum, by semi-rotatory movements it can be gradually insinuated into the lowest part of the sigmoid flexure. The most satisfactory results are obtained by holding the vessel containing the fluid about two feet above the patient, and after a small amount of the water has been allowed to trickle into the gut to gradually raise the reservoirs to the height of three feet. When the bowel is fully distended slight additional pressure may be exerted by raising the receptacle a few inches higher still, which in most cases will be followed by the immediate and forcible expulsion of both catheter and fluid. The temperature of the water should vary with the intensity of the fever and the resistance this offers to the antifebrile process. Water cooled by ice to a temperature of from 52 to 55 deg. F. is used in cases of mild pyrexia. When the temperature of the patient indicates hyperpyrexia, the temperature of the water is reduced to 43 deg. F. The amount of water to be injected into the gut may vary from one to two or even three pints.

